# New Drugs and Preparations

**Published:** 1894-06-02

**Authors:** 


### New Drugs and Preparations.

[We shall be glad to receive, at our Office, 428, Strand, London, W.C., from the manufacturers, specimens of all new preparations and drugs which may be brought out from time to time.]

### SULFONAL.

Kast (Archiv f. expt. Path. xxxi. 69) reviews the results of four years' experience of sulfonal since its first introduction into medical practice by Baumann and himself. He states that certain points could be settled only after very considerable experience of its use, namely—(1) A more precise knowledge of its special indications and contraindications; (2) a knowledge of possible dangers from its long-continued administration; (3) the most suitable dose for occasional or habitual use. The drawbacks and objections which have been instanced against it he characterises as of very unequal importance. For example, its failure to produce sleep in a certain number of cases it shares with all other hypnotics, the same being true of an occasional exciting instead of a calming effect, while "deferred" action can be to a very large extent overcome by taking care to dissolve it as far as possible in hot water. Moderate doses taken occasionally are quite harmless, and even large doses, 60-90 grains, although they may produce giddiness, ataxia, ptosis, paralysis of vision, and other disagreeable symptoms, are not dangerous. Doses of 150 grains, even 190 grains and more, have been taken without proving fatal. On the other hand its continued and habitual use is now known to be attended with very grave consequences. (1) There occur vomiting, diarrhœa, and severe constipation; (2) Ataxia, weakness in limbs, ptosis, ascending paralysis; (3) Ischuria and oliguria, albuminuria, and a cherry red colour of the urine due to hæmatoporphyrin. Death has been recorded in a considerable number of cases, chiefly asylum patients who had taken the drug for long periods. Experiments on animals showed that it caused hæmorrhages and other structural changes in the kidney: but only when taken in large quantity. To avoid these dangers, Kast advises: (1) That the most suitable hypnotic dose for males is about 30 grains, for females 15 grains. As it is slowly excreted, these must be also regarded as the maximum daily amounts which should be administered. For lunatics larger doses are allowable, and are required. (2) During its habitual use, it should be intermitted from time to time for one or several days, as may seem necessary. (3) Digestive troubles of any kind, and especially the red colour of the urine, are signs of intolerance on the part of the organism, and indicate cessation of the drug. (4) With these precautions, and in suitable doses, it is quite safe and harmless. Gonzales ("Archiv. di Farmacol e di Terap.," 1893), from the result of experiments on animals, advises the combined use of sulfonal and morphine as safe, and calculated to give more satisfactory results as a hypnotic than either drug separately. There is no depressing action on the heart, and pain is controlled.

### EUCALYPTUS OIL.—FATAL CASE OF POISONING.

Neale reports (*Australian Medical Gazette*, April, 1893) a fatal case of poisoning with eucalyptus oil in a boy aged 10. So far it is the only case of the kind on record, although an ounce is stated on one occasion to have accelerated death in an adult. The boy took a little over half an ounce at nine p.m. as a preventive against "catching a cold," from which other members of the family were suffering. In a few minutes he was gasping for breath, and vomited. This gave some relief, but gradually the dyspnœa increased again, and he died fifteen hours after ingestion of the oil. There was no purging, no convulsions, and he was sensible until within an hour of his death. Post-mortem examination showed the stomach to be much distended, the outer surface white, the inner white, and thickened and puckered as if painted with a mild solution of carbolic acid; the pleural cavities contained a quart of serous blood, but there was no lymph on the pleuræ nor thickening, both lungs collapsed, firm and bloodless. Otherwise the organs were healthy. He was not treated by a medical man apparently. The author states that serious symptoms with dyspnœa, have often followed the dose of one drachm, but always resulting in recovery.

